# Emotion Recognition on Edge Devices: Training and Deployment

**DOI:** 10.3390/s21134496

**Published:** 2021-06-30

**Authors:** Vlad Pandelea, Edoardo Ragusa, Tommaso Apicella, Paolo Gastaldo, Erik Cambria

**Affiliations:** 1 School of Computer Science and Engineering, Nanyang Technological University, 50 Nanyang Ave, Singapore 639798, Singapore; vlad.pandelea@ntu.edu.sg; 2 Department of Naval, Electric, Electronic and Telecommunications Engineering, University of Genoa, 16145 Genova, Italy; edoardo.ragusa@edu.unige.it (E.R.); tommaso.apicella@edu.unige.it (T.A.); paolo.gastaldo@unige.it (P.G.)

**Keywords:** emotion recognition, embedded systems, deep learning

## Abstract

Emotion recognition, among other natural language processing tasks, has greatly benefited from the use of large transformer models. Deploying these models on resource-constrained devices, however, is a major challenge due to their computational cost. In this paper, we show that the combination of large transformers, as high-quality feature extractors, and simple hardware-friendly classifiers based on linear separators can achieve competitive performance while allowing real-time inference and fast training. Various solutions including batch and Online Sequential Learning are analyzed. Additionally, our experiments show that latency and performance can be further improved via dimensionality reduction and pre-training, respectively. The resulting system is implemented on two types of edge device, namely an edge accelerator and two smartphones.

## 1. Introduction

Sentiment analysis and emotion recognition are key tasks to build empathetic systems, as well as to enable intelligent behavior driven by the user’s emotions [1,2,3]. With the advent of pervasive computing the challenge of migrating solutions that were originally designed for servers to edge devices becomes increasingly important and paves the way for novel applications and for the upgrading of existing ones.

Fine-tuning models on user’s devices using user’s data is of primary importance for multiple reasons. First, smart devices produce an amount of data that cannot be processed using server-based solutions. Even with the development of super-computers only a small portion of the data produced can be processed [4]. For this reason, it is unrealistic to build user-taylored solutions without employing edge devices. It is important to stress out that this bottleneck will drive most of the choices in the rest of the paper. Second, the so-called subjective perception problem [5] affects all the general purpose solutions for sentiment analysis. Indeed, building a general purpose sentiment model implies an underlying consensus of the users regarding topics like religion, politics, and many other controversial topics. Obviously, building a model for each user using cluster based solutions is prevented by the computational bottleneck discussed in the first point. Third, cloud computing is subject to a weak and still under-development regulation. Accordingly, privacy concerns arise especially when one recalls that emotion recognition and sentiment analysis allow for user profiling [6,7].

Deep learning paradigms prove effective in gauging the sentiment of users, but resource-constrained devices cannot support the training process, and even deploying trained models on embedded systems still remains a challenging task. Thus, large pre-trained models [8,9,10] that can be fine-tuned for downstream tasks are not apt to be deployed and customized for the end-user due to their large computational power requirement. Most of research activity then tackles the deployment problem using a high power machine that perform a set of optimization during and after the training phase. Shrinking the models using pruning and compression strategies [11] are appealing solutions. In the later years knowledge distillation has been largely employed to embed the high quality feature extraction capability of large size models on small size networks [12]. Finally, one can recall quantization techniques that, when supported by appropriate hardware resources, can reduce memory requirements, inference time, and energy consumption [13]. Despite their effectiveness, all these techniques assume a large set of hardware resources during training and their deployment cannot be supported by most edge devices.

At the same time, the larger models can be exploited as high quality feature extractors as the inference phase of these models is orders of magnitude faster than their training. Indeed, one can rely on the standard fine-tuning paradigm with only the last layer, i.e., the classification layer of the network, trained on the novel problem. Obviously, one can also replace the last layer with a standard classifier. As a major advantage, the training problem can be deployed on constrained devices.

In this paper, thus we demonstrate that training of models at the edge is feasible and can maintain good performance while attaining real-time inference and extremely fast training time without the usual assumptions on the availability of computational resources, such as powerful servers or distributed computing reliance. The paper benchmarks a set of state-of-the-art solutions for feature extraction, and proves that a two stage fine-tuning strategy can lead to efficient yet effective predictors. As a major result, the paper proves that classifiers that only require the training of a simple linear separator lead to satisfactory generalization performance for the target problem. Thus, while we do not propose novel architectures or algorithmic innovation aimed at advancing the state-of-the-art in emotion recognition, we show that the fast and efficient training procedure in this paper can be easily deployed on edge devices. In addition, empirical studies concerning dimensionality reduction, task-specific pre-training, and tuning on single user data are presented. Finally, the eventual training pipeline is deployed on a edge accelerator, namely Nvidia Jetson Nano, and on two smartphones. Code for the test on smartphones available at: https://github.com/SEAlab-unige/Sensors-2021-Emotion, accessed on 29 June 2021.

### Contribution

The main contribution of this work is the design and deployment for edge devices of competitive emotion recognition solutions, that is, solutions that maintain good generalization performance while allowing for fast on-device training and real-time inference. Accordingly, a novel training pipeline suitable for edge devices is proposed. The pipeline is novel in that the fine-tuning process leads to satisfactory performance using simple and fast training procedures based on linear separators. This result is due to the excellent feature extraction capabilities of transformer models. Two categories of devices are considered for the deployment of the proposed solution: first is a high performance system for embedded deep learning applications, second is the class of smartphone devices.

Our experiments demonstrate the feasibility of the proposed models and the trade-offs in terms of accurate emotion detection and computational requirements as assumptions and design choices vary. Additionally, as minor contributions we present empirical studies that demonstrate that:

1. pre-training on a different dataset for emotion recognition can improve performance at no additional cost after deployment;
2. dimensionality reduction can achieve reasonable trade-off between performance and computational requirements;
3. limited single-user data maintains comparable performance to a larger number of data samples.

## 2. Related Work

There is a vast literature on emotion recognition from video and user-generated speech [14,15,16,17,18]. Similarly to other natural language processing (NLP) tasks, recent years have seen large transformer based models being increasingly used as they are able to effectively model contextual data and extract high quality features [19,20]. At the same time, the challenges posed by the computational load of these models have been previously acknowledged by proposing variants of the original Bidirectional Encoder Representations from Transformers (BERT) [8]. Ref. [21] proposed MobileBERT, a thin version of BERT${}_{LARGE}$. Refs. [22,23,24] proposed compact models trained through knowledge distillation. Ref. [25] used grouped convolutions to accelerate the inference process.

Beyond the comparison of these compact architectures inspired to their larger counterparts, few works that empirically study or propose different approaches to tackle the limitations posed by embedded devices have also been published in related task such as polarity detection from visual data [26,27], speech recognition [28] and conversational agents [29]. In addition, the trade-off between computational cost and generalization performance for sentiment analysis was studied in [30,31], but the focus was not on the deployment of the solution.

Training deep learning models on single edge devices is a relatively new topic because most work is still spent on the implementation of the inference phase [32]. Indeed, significant efforts are spent in the definition of novel hardware architectures that support energy efficient inference phases [33]. The most active research line for training on constrained environment envision an edge server that coordinates the training process with multiple edge devices [34]. This paradigm augments computing resources using multiple nodes. In this setting, communications bandwidth, updates frequency, and workload split among different nodes play a major role. Federated learning handles effectively the aforementioned issues [35,36]. The basic flow starts with a trained global deep learning model. Each device receives a copy of the trained model. This global model is then updated locally on each device with local data. Then, the new models are sent back to the server and combined. Other interesting options to share the work load on different nodes came from distributed training strategies inspired by online distillation [37]. However, all these solutions assume a sufficient support from hardware resources. This assumption is rarely met by most of the constrained devices typically employed in real-world applications. Indeed, incremental learning strategies were tested because they reduce the burdensome cost of batch learning [38,39].

To perform training on a single device we rely on the standard feature extractor-classifier paradigm [40]. For the classifiers we explore models based on a specific kind of Single-Layer Feed-Forward Neural Networks(SLFNs). Actually, in recent years most of the work concerning SLFNs targets random based solutions [41]. Among the existing paradigms for Random Based Networks (RBNs) we can mention Random Radial Basis Functions [42], Random Vector Functional-Link (RVFLs) [43], Extreme Learning Machines (ELMs) [44], and Weighted Sum of Random Kitchen Sinks [45], that offer interesting opportunities. For convenience, hereinafter we will refer to Random networks as ELM, because it features a vast, long-standing literature within the existing RBN approaches. These paradigms features fast learning and an efficient forward phase due to the fact that the hidden parameters are not tuned during the learning process. The literature shows that SLFNs [41,46] offer a viable solution for low-power, resource-constrained digital implementations of the inference function [47,48], even making the on-device support of the training process possible on dedicated systems on chip [49,50,51,52]. In addition, SLFNs can be straightforwardly extended to Online Sequential Learning [53].

Unlike previous approaches, the method proposed in this paper allows for direct on-device training in addition to real-time inference of emotion recognition systems. In practice, we admit only learning paradigms with a training strategy that coincides with the training of a linear separator. From a pure mathematical point of view, fully-trained shallow networks have the same generalization capabilities of deep learning approaches [54]. However empirical results proved that in real world applications deep learning networks converge to better solutions.

## 3. Materials and Method

The primary objective of this work is to tackle the understudied problem of deploying emotion recognition on constrained devices, including the training phase. This is a difficult problem as training directly on a resource-constrained devices requires additional precautions to efficiently balance between performance, in terms of accuracy or F1 Score, and computational cost. To do so, we propose a pipeline that combines state-of-the-art solutions for feature extraction in NLP with hardware-friendly methods for emotion classification.

**Figure 1:**
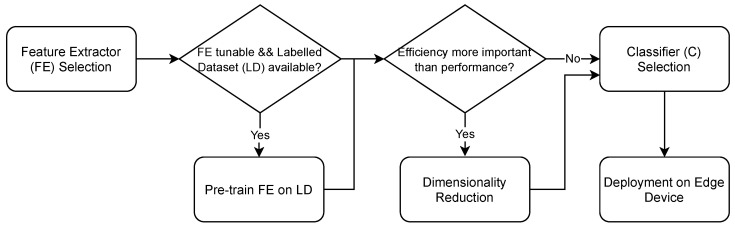
Pipeline of the system.

A key observation is that while executing the training phase of deep models on resource-constrained devices is not practical, it is possible to exploit their inference phase to extract high-quality features from the raw data, that can then be fine-tuned via much smaller and simpler models whose training phase can be reasonably executed directly on such constrained devices. Because the deep models cannot be fine-tuned, it is of utmost importance to select models that can extract features conducive to good performance without being trained on the specific data. Moreover, additional steps can be taken to increase both performance and efficiency. Figure 1 illustrates the pipeline, and in the following we go into greater detail as concerns our design choices. Given a new data point *s*, we also illustrate the process it goes through:

1. Feature Extractor (FE) Selection: a sensible choice for the feature extractor is found in the large models based on the transformer architecture [55] that have been trained on huge corpora and shown to achieve impressive results in various tasks once fine-tuned. In fact, these models have been proven to be able to extract high-quality features that can adapt to a range of different tasks with little fine-tuning. Despite requiring only a brief fine-tuning phase as opposed to training from scratch, when considering the additional constraints of embedded and portable devices, this is still too demanding. Therefore, as will be shown in our experiments, some loss in performance is inevitable when the fine-tuning of the entire architecture does not take place, however on-device training becomes possible.
  When a new data point *s* is collected, its features are extracted via FE:
  (1) $$\begin{array}{c} h_{s}=FE\left(s\right) \end{array}$$
2. Pre-training on Labelled Dataset (LD): an optional step that can increase performance is, when the feature extractor is tunable, pre-training on a labelled dataset from a similar domain. While the available pre-trained transformer models are already pre-trained, this is done on quite general language modeling tasks. Thus, a further pre-training on data that more closely resembles the end application can prove beneficial. At the same time, doing so could aggravate the subjective perception problem.
3. Dimensionality reduction: if efficiency in terms of latency and/or training time is crucial, such efficiency can be further improved at the cost of some performance. A standard method to do so is reducing the input data dimensionality. This often can lead to performance degradation as information may be lost in the process, however the efficiency generally increases.
  The features $h_{s}$ undergo a transformation via a dimensionality reduction method DR:
  (2) $$\begin{array}{c} h_{s}^{dr}=DR\left(h_{s}\right) \end{array}$$
  For example, with Principal Component Analysis (PCA):
  (3) $$\begin{array}{c} h_{s}^{dr}=W^{T}h_{s} \end{array}$$
  where W is the matrix of eigenvectors of the PCA.
4. Classifier (C) Selection: the previous steps refer to the pre-processing of raw data in order to obtain high-quality features on which to fine-tune a classification model C. C should be a classifier satisfying the constrained environment assumption, thus, depending on the device, most modern deep learning based models may not be suitable. We pick simple neural models, such as SLFNs, with a training that coincides with the tuning of a linear separator. In practice, the tested solutions range from random based solutions to simple perceptron-like networks. These models feature a set of advantages when training is performed directly on edge devices. First, with certain cost functions, the training procedure admits a closed form solution. Second, the optimization process is convex and the computational cost is small. Third, the number of hyper-parameters is small, as a consequence of which little human intervention is needed.
  The prediction is obtained via the classifier as:
  (4) $$\begin{array}{c} y_{s}=C\left(h_{s}\right) \end{array}$$
  where we use $h_{s}$ for convenience. $h_{s}^{dr}$ if dimensionality reduction was applied.
  For example, in the case of a SLFN, random based solution, we would have:
  (5) $$\begin{array}{c} y_{s}=softmax\left(W_{o}\sigma \left(W_{h}h_{s}+b_{h}\right)+b_{o}\right) \end{array}$$
  where $W_{o}$ and $b_{o}$ are trainable parameters, $W_{h}$ and $b_{h}$ are initialized randomly, and σ is an activation function.
5. Deployment on Edge Device: after the deployment, C can be fine-tuned as new batches of data are collected. The frequency of the updates should depend on the end-application as well as on C. For instance, in the case of OS learning solutions, more frequent updates are possible.

Algorithm 1 briefly recaps the pipeline.

The following subsections detail the key components of the proposed solution.

### 3.1. Feature Extractor

Throughout this paper we explore a range of solutions, including near-state-of-the-art solutions as concerns transformers used in NLP, for emotion recognition from conversational data. In particular, we experiment with four transformer-based models, to act as feature extractors, that differ in the pre-training strategy, number of layers, layer width, and embedding size. In this section, we briefly describe each of them:
**Algorithm 1:** Complete procedure outline.**Input**:-(optional) Labelled Dataset (LD) from similar domain as end application;-Feature Extractor (FE);-Edge device;**Pre-deployment**:-Place a standard classifier (C) satisfying the edge device constraints on top of the FE;-(optional) Further train the FE on LD;**After deployment**-While *(condition to update)* not met:-Collect data point *s*-Extract features $h_{s}=FE\left(s\right)$-(optional) Apply dimensionality reduction $h_{s}^{dr}=DR\left(h_{s}\right)$-Use classifier C to get the prediction $y_{s}=C\left(h_{s}\right)$, or $y_{s}=C\left(h_{s}^{dr}\right)$-Fine-tune C on the new data points collected

1. RoBERTa: Robustly Optimized BERT [9] is an approach that modifies BERT pre-training by, among others, using more data and removing the Next Sentence Prediction (NSP) objective. It was shown to significantly benefit from these changes and achieve state-of-the-art performance in several tasks. Like BERT, two configurations differing in the model size are released. For our experiments we use the BASE version that uses a hidden layer size of 768. This model presents the same computational complexity as BERT once pre-trained. It is the most computationally intensive model that we experiment with in this paper. The estimated model size as imported without further optimization is 499 MB.
2. MobileBERT: MobileBERT [21] reduces the computational load of BERT by equipping the model with bottleneck structures. The original paper shows that the model is up to 5.5 times faster than the BASE version of BERT. The output size of this model is 512. Unlike other compact versions of BERT, MobileBERT maintains the depth while only reducing the width, which may allow more representation power that could in turn prove useful when fine-tuned. The estimated model size as imported without further optimization is 99 MB.
3. BERT-Medium: BERT-Medium [24] is another model that aims to reduce the computational load of BERT, via a three stage approach, including pre-training of compact models, distillation from a larger model and optional fine-tuning. We use two versions that differ in model size. The first one has 8 layers and a hidden size of 512. This model has about 38% of the parameters of BERT and achieves a speedup of 3.64 [24] with respect to BERT${}_{LARGE}$. The estimated model size as imported without further optimization is 167 MB.
4. BERT-Tiny: Similar to BERT-Medium, this compact version of BERT differs in the number of transformer layers, featuring 2 of them, and in the hidden size equal to 128. This is the smallest model in our experiments with only 4% of the parameters of BERT and a speedup of 65.24 with respect to BERT${}_{LARGE}$. The estimated model size as imported without further optimization is 18 MB.

### 3.2. Classification Model

The selection of the classifier is one of the most critical steps in the system. In fact, training without fine-tuning the feature set implies that the eventual classifier is strong enough to learn rules from sub-optimal features. This requirement clashes with the limited computing resources in that accurate approaches usually stand on demanding training processes. In order to limit the search space we opted for solutions based on shallow neural networks because they offer a favorable trade-off between generalization performance and computational cost. For each of the feature extractors we employ, three different setups are considered.

1. Linear: a linear layer is placed on top of the frozen FE. The FE parameters are not updated during backpropagation, which greatly accelerates the training process at the cost of less problem-specific features, and thus a drop in performance is expected. Indeed, this solution underlies the strong assumption that different classes, in the general purpose feature space, are linearly separable.
2. ELM [56]: ELM is a training paradigm for single hidden layer neural networks where the parameters of the hidden layer are not tuned during the training phase. In theory this model has universal approximation capabilities like SLFN, but the training phase involves the same computational cost of a linear separator. In practice, ELM training allows fast non-iterative procedures, including closed-form solutions. At the same time, in the general case it requires a Least Squares Loss, which might lead to performance degradation on classification problems.
3. OSELM: Online Sequential ELM (OSELM) is another implementation of ELM that is suitable for online learning scenarios as it can update its parameters with chunks of data at a time, including chunks of a single data point. Under the assumption that the first batch of data contains no less data points than the number of hidden nodes, OSELM can achieve the same performance as ELM [57].
  OSELM [53] features two appealing peculiarities for our target applications. Firstly, training using chunks of data reduces memory requirements. Secondly, it allows updates of the model every time that novel data are available.

### 3.3. Hardware Setup

The selection of the hardware device is driven by two hard constraints: (1) the system should support the forward phase with small latency. When a new sample is available the system prediction should be provided within a few milliseconds; (2) the system should support the training of the classifier C when new data are available. Obviously, these two hard constraints are paired with the requirements of low power and small size. In practice, we would like to solve this problem with the smallest and less power hungry system possible.

The market offers several edge devices suitable for the deployment of deep learning models. These devices differ in power constraints, dimension, memory size, overall architecture, and software support. Here we explore two classes of solutions. The first solution is a high performance system for embedded deep learning called Jetson Nano. The second solution is represented by smartphone devices.

Jetson Nano is a System On Module (SOM) composed of an ARM A57 quad-core, a 1.43 GHz CPU, and a Maxwell 128 core GPU equipped with 4 GB of RAM memory. The device hosts an OS derived from Ubuntu 16 that enables a high level of control of hardware resources. The entire Jetson Nano module measures 7 cm × 4.5 cm.

Nvidia proposes two optimized configurations of the hardware resources of Jetson Nano that can be selected from a software interface, namely 5 W and Max-N. Informally speaking, 5 W is the low power mode. In this configuration, only two cores of the ARM A57 are power supplied, and the clock frequency is limited to 0.9 GHz. Moreover, the clock frequency of the GPU is bounded to 0.64 GHz. The Max-N mode turns on all the 4 cores and sets the frequency of the CPU to 1.5 GHz. GPU clock frequency is 0.92 GHz.

Nvidia provides a dedicated SDK and TensorRT for the optimization and quantization of deep learning models. The software engine uses native TensorFlow. Accordingly, almost all the layers are supported on these devices. This aspect allows deploying recent solutions that are not supported by most of the embedded systems relying on custom inference engines. This offer high computing power with small form factor. However, power consumption might represent a critical issue in Jetson Nano. This device could represent a valuable option in two scenarios: (1) applications such as smart-houses where a power source is available; (2) embedded in portable devices with an ad-hoc battery management strategy that powers on the devices only when needed.

The second class of devices are the smartphone processors. These systems represent a reliable solution due to the excellent ecosystems with high-level of integration with standard deep learning tools. We consider only smartphone processors, avoiding the use of dedicated GPUs and NPUs that are efficient but non standard. Two different solutions have been tested, namely, Snapdragon 765G and HiSilicon Kirin 655, to demonstrate that our solution can be deployed to systems with different processing capabilities.

Among the available libraries for deployment we selected TFLite that supports the majority of the deep learning layers. In addition, one can setup the tool for post training quantization of the deployed model.

## 4. Generalization Performance Results

To evaluate the effectiveness of the proposed solution, an experimental setup simulated a real use-case. The experiments were divided in 4 subsections, one for each topic previously introduced. In Section 4.1 the generalization performance of the proposed approach is compared against two baseline solutions, including the end-to-end counterpart. In Section 4.2 we explore the benefits of task-specific pre-training. In Section 4.3 we study the use of input dimensionality reduction to reduce the overall computational load. Finally, in Section 4.4 we show the performance of the system on limited single user data.

The experiments make use of two datasets. The first, MELD [58], is a dataset containing conversations from the TV series Friends. There are six main speakers and each utterance is annotated with one of seven categorical emotions, namely Anger, Disgust, Sadness, Joy, Neutral, Surprise and Fear. We use the data splits provided. In particular, there are 9989 utterances in the training split, 1109 in the validation split and 2610 in the test split. All of the results reported are on the test set. The same subjects appear in all of the splits. The average utterance length in the dataset is 8 words. For convenience, we report the distribution of emotions of this dataset in Table 1.

In Table 2 we show a few samples from this dataset together with our system’s predicted label as per the single user training experiment described in Section 4.4. Notice how, even when emotion recognition systems are wrong, the predicted label can actually be reasonable, as it happens in the second and third samples. Other times, like in the fourth sample, additional context is needed to detect the correct emotion as the textual information may not suffice.

IEMOCAP [59] also contains annotated conversations, between pairs of two speakers. Conversations are acted and the actors either improvise or follow a script depending on the particular conversation. Like [60], we also use the six most common emotions, namely happy, sad, neutral, angry, excited, and frustrated, and the same data splits. In the future, however, we plan to use the Hourglass of Emotions [61] as categorization model. The distribution of the emotions in the data used is reported in Table 3.

Training Setup

Throughout the experiments, for the fine-tuning of the transformer and the Linear and Hidden solutions we employ the Cross Entropy loss function during training, whereas ELM and OSELM use a MSE loss. Model selection is performed for each solution varying the L2 regularization factor and number of hidden units, where applicable. In particular, for the Hidden solution the set of hidden units is {10,15,25,50,100,200,500,1000} and the set of L2 regularization coefficients {0.0,0.001,0.003,0.01,0.03}. We use the Adam optimizer with learning rate 0.0001 when training the Linear and Hidden solution on all users, and SGD with learning rate 0.01 when training on single user data and to measure on-device training time. Batch size is set to 64. All the utterances are padded to 32 tokens.

We consider, as baselines, two solutions that do not satisfy our training requirements assumptions:

1. The entire model is fine-tuned: a linear layer is placed on top of the FE and the whole model is fine-tuned end-to-end. This is expected to produce the most accurate result as the FE can adapt to the task and dataset, and extract features that are highly problem-specific. At the same time it is also the most computationally intensive one as we need to backpropagate the error, during gradient descent, through all of the layers. This solution is intended as an upper-bound reference to compare performance in terms of accurate emotion recognition, and is not generally applicable in edge devices.
2. Hidden: a single hidden layer feedforward network with tanh activation is placed on top of the frozen FE. This allows for non-linear transformation of the features extracted by the FE. Since these features are not problem-specific, this solution is expected to increase performance with respect to a linear transformation, at the cost of fine-tuning an additional layer and performing a non-linear operation.

### 4.1. Generalization Performance

Table 4 reports the performance in terms of weighted F1 score of the various architectures. The headers in the first row denote the transformer used. The second row represents the solution in which the transformer is fine-tuned using backpropagation (BP), whereas in the subsequent rows the transformer is frozen and acts only as a feature extractor. In particular, the third row (Hidden) reports the results for the second baseline solution. The rows from 4 to 6 identify the classifiers introduced in Section 3.2.

Unsurprisingly, the larger models tend to perform better, and fine-tuning always improves performance significantly at the cost of drastically increased training time. Moreover we find that the Linear solution tends to outperform ELM and OSELM, which is due to the more suitable cost function for the classification task. A hidden layer proves not to significantly aid performance in the case of RoBERTa, likely due to the higher quality of the features extracted by this model when it is frozen. It is worth to recall that not only training time but also memory constraints prevent fine-tuning of all network parameters in embedded devices.

For additional comparison, state-of-the-art method COSMIC reports an F1 score of 0.6521 [60]. However this method uses a commonsense knowledge extractor trained on large knowledge bases. As reported in [60], RoBERTa achieves similar performance (0.6202) to what we report here. Combining RoBERTa with more context-aware methods such as DialogueRNN, slightly increases the performance (0.6361). Both COSMIC and DialogueRNN utilize additional context and involve more computations. Instead, in this paper we only train simple classifiers such as SLFNs.

### 4.2. Feature Extractor Task-Specific Pre-Training

While the feature extractors are pre-trained via methods such as Masked Language Modeling [8], that are self-supervised and represent a solid starting point to a wide range of NLP tasks, it might prove useful to further pre-train in a supervised setting that more closely resembles the final deployment condition. To this end, we experiment with pre-training on a different dataset, IEMOCAP, that is labelled for emotion recognition, albeit using slightly different emotion categories.

Although both datasets are scripted to some extent, the style of the conversations that take place is largely different, thus representing a realistic scenario to improve the performance for the application’s end-users.

When pre-training on IEMOCAP, we perform early stopping at about 80% of the final performance, as from our experiments we find that carrying out the pre-training up to the optimal point on one dataset is not conducive to increased performance on a different one. We surmise that this is due to the feature extractor slowly moving from being task-agnostic, to task-specific, to dataset specific.

Table 5 shows the results for this experiment in terms of F1 score. The headers of the column pairs denote the base transformer architecture, the sub-headers denote the regular and the pre-trained variants respectively, the rows denote the top-structure that is fine-tuned. We find that in almost all the cases the pre-training proves to considerably enhance performance. Again, Hidden is the baseline solution. Another beneficial effect of this is that the increased quality of the features extracted through the pre-trained variant often leads to a flattening of the differences between the top-structures thus enabling more efficient solutions without sacrificing as much in terms of accurate emotion recognition [62].

### 4.3. Dimensionality Reduction

When the goal is to obtain efficient solutions in terms of latency and/or memory consumption, it is often worthwhile to explore dimensionality reduction techniques. PCA is one such technique, and the one that we adopt in this work. In particular, we reduce the dimensionality to 50% and 25% of the original one for BERT-Tiny, and to 25% and 12.5% for BERT-Medium.

Table 6 shows the results in terms of F1 score. The first column identifies the feature extractor and the output dimensionality after PCA. When PCA is not specified, the original output size is maintained and placed after the name of the model. The remaining columns remark the classifier that elaborates the extracted features.

As can be seen from Table 6, reducing the input data size leads, in most cases, to a noticeable drop in performance and thus its use should be carefully evaluated based on the application requirements. For BERT-Tiny we can reduce the feature set size by 50% with an average absolute loss in performance, across the models, of 0.725% and by 75% with an average loss of 1.875%. Similar results can be observed for BERT-Medium.

### 4.4. Performance under Limited Data Assumption

A realistic use-case scenario for embedded systems often involves handling solely the data pertaining to a single user. Moreover, such data can often be scarce. To consider this setting we design an experiment in which the system is trained and evaluated on each user in our dataset separately. Additionally, we cover three cases that differ in the amount of data available. The dataset contains six frequent users, whereas the others are aggregated under the “*Others*” label in the original annotation. We only use the six most frequent users.

The results of this experiment are reported in Table 7. Here, each row denotes an user and each column denotes the amount of data. Again, all results refer to the weighted F1 score. “All” indicates that all of the data available for the user, 1200 to 1500 data points depending on the user, is utilized. For validation and testing we utilize the same splits as in the previous experiments (limited to the user we train on). The scores reported refer to the BERT-Tiny model with IEMOCAP pre-training and PCA with 32 components. The classification layer is the Linear one. As can be seen, in most cases 500 data points can maintain a satisfactory performance, whereas in some cases 200 data points may lead to a steeper drop.

## 5. Hardware Deployment Results

We designed tests dedicated to measure the performance of the proposed solution when deployed on the embedded devices. We propose two tests: (1) feature extraction where we measure the time to process one sentence; in fact, this test also measures the trend for the prediction time because the additional cost introduced by the linear separator during the forward phase is negligible. (2) training time where we measure the time to train a linear separator on the device.

### 5.1. Feature Extraction

The test encompassed two models, BERT-Tiny and BERT-Medium. We measured the time for the elaboration of a sentence, starting from the tokenization until the end of the forward phase of the deep learning model. The system processed the sentences individually, i.e., the batch size was fixed to 1 and the utterances were padded to 32 tokens.

The first deployment pipeline involved the Jetson devices. The pipeline started from the trained model described using Keras API. We converted the model in TensorFlow and we froze it. Thus, the frozen model coincides with the original model where all the software structures supporting the training phase were removed. The frozen model was then optimized for Jetson Nano using the TensorTRT tool (https://developer.nvidia.com/tensorrt, accessed on 29 June 2021). The output is again a TensorFlow frozen graph where the computed layers are replaced with optimized versions.

TensorTRT can adopt different data sizes when deploying a network on Jetson Nano: standard floating-point representation (FP32) and half-precision floating point (FP16).

Table 8 shows the summary results of the experiments for Jetson Nano. The rows show the working mode of the device. The first and second columns identify the inference performed using the BERT-Tiny model with 16 and 32 bits quantization, respectively. The third and fourth columns reports the same information for the BERT-Medium model. Each cell contains the average inference time (in ms) for a sentence measured over 80 random sentences extracted from the MELD test set. Between brackets we show the standard deviation.

Numerical results point out some interesting outcomes. First, both the configurations ensure the elaboration of a sentence in less than 50 ms. Accordingly, the device can extract features in real time. Second, quantization in 16 bit does not yield a significant advantage in terms of computing time. This is likely due to the fact that the models are relatively small.

The second set of experiments considered the smartphones. The pipeline started from the Keras model converted into TFLite graph using Keras API. TFLite supports different quantization levels. In this paper, we consider three quantizations: FP32, FP16 and INT8. The host devices were a Snapdragon 765G, and a HiSilicon Kirin 655. All versions are deployed on actual devices through an Android app. The app performs the inference phase using only the CPU and collects mean and standard deviation of the forward phase time, which also includes the tokenization process.

Table 9 inherits the structure from Table 8. The difference is that the rows stand for different chipsets and the groups of columns contain an additional column denoting INT8 quantization.

Experiments highlight a few interesting outcomes. First, the BERT-Tiny model, optimized with TFLite, is faster than the fastest model on Jetson Nano. However, the BERT-Medium model introduces a larger latency. This is due to the trade-off between memory fetch and capability of parallel computing. The empirical results suggest, as obvious, that when model size grows Jetson Nano can handle the floating point operations more efficiently. On the other hand, for small size models TFLite proves more efficient than TensorRT in data transfer, that becomes the bottleneck of the computational pipeline. A second outcome is that, as in the case of Jetson Nano, FP16 quantization provides a negligible speedup. Meanwhile, INT8 inference ensures a faster inference phase.

**Figure 2:**
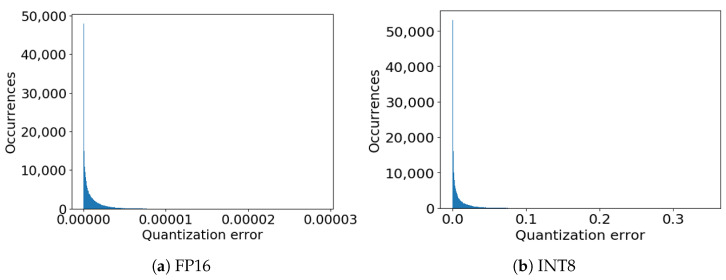
Rounding error distribution on MELD test set using TFLite interpreter.

Despite being convenient in terms of latency and memory requirements, the quantization introduces a rounding error. Figure 2a shows the histogram for the quantization error introduced by FP16 and Figure 2b remarks the error for the INT8 case. In both the cases the error is computed against the FP32 representation. The histogram takes into consideration the quantization error for all the features and all the data in the MELD test set:

(6) $$e={\left(h_{i,j}^{FP32}-h_{i,j}^{Quant}\right)}^{2}$$

where $h_{i,j}$ refers to the *j*-th output of the feature extractor for the *i*-th datum. The superscrpt denotes the representation. FP32 is the reference. Quant stands for FP16 and INT8 for the two experiments.

The results show that for FP16 the quantization error is negligible, i.e., the values are lower than $10^{-5}$. For INT8 the average error is small. However in a few cases, the measured difference is in the order of 0.1. Indeed, this value can introduce small changes in data representation. However, the classifier *C* is trained directly on the new quantized representation. Accordingly, the effect for the generalization performance are very limited. To verify this, we experiment with the Linear model on top of BERT-Tiny, where the features are extracted with the three different representations FP32, FP16 and INT8. We find that FP16 does not lead to any noticeable drop in F1 score and, similarly, INT8 leads only to a 0.05% decrease in performance. Therefore, quantization proves to be an effective strategy to reduce resource consumption.

### 5.2. Linear Separator Training

The second set of experiments encompasses the training of a linear separator using the embedded devices. This setup proves empirically that the selected embedded devices can support the proposed training phase.

The experiments analyze a worst-case configuration where a linear separator is trained using gradient descent. This setup has an higher cost with respect to OSELM training. However, it is more general because it can be applied to a wide set of cost functions.

The setup aims to test the computational cost. The simulation employs random data, ensuring that the solution does not converge in a small number of iterations. The number of iterations was set to 100 epochs, as all of the models trained on complete single user data in Section 4.4 converged in less than 100 epochs.

The code for the tests on the Jetson Nano was developed in Python using sklearn module utilities. This solution was selected because it can be easily interfaced with Tensor RT and TensorFlow and offers optimized implementation of the learning algorithms.

Table 10 shows the results concerning the simulations on Jetson Nano. The first column identifies the working mode of the Jetson device. The second column denotes the number of features. The range of values tested includes the number of features extracted by BERT-Tiny, BERT-Medium, as well as the variants where PCA is applied. The remaining columns stand for the number of training data samples. We use 1300 to represent the case in which all of the user’s data is available. Each cell shows, in milliseconds, the average time required to complete 100 epochs using SGD. The average was computed over 30 random extractions of the training data. Between brackets we report the standard deviation.

The results unveil that Jetson Nano can handle the training procedure of the model in less than a second. When Z=200 the training time for a dataset with 128 features is smaller than the one for 64 feature. This could be attributed to the trade-off between data transfer and parallelization capabilities. Except for this particular case, the measures confirm that training data and features set size play a major role in determining the training time.

The same set of experiments was performed on smartphones. The experiments were performed using an app written in Kotlin.

Table 11 inherits the structure from Table 10. The only difference is that, in this case, the first column denotes the chipset.

The results confirm that the smartphones can host the training process. Indeed the worst time measured was around 7 s. This time refers to a training that should be performed only periodically and that does not affect the inference time at all.

## 6. Conclusions

In this paper, we explored the combination of various state-of-the-art architectures for feature extraction in NLP with simple and fast classifiers, whose training strategy is no more demanding than a linear separator, for emotion recognition. Further, we proposed a pipeline to optimize the system and deploy it in a constrained environment. Experiments demonstrated that the final system can achieve real-time inference and fast training, for single user data, on two classes of edge devices while retaining satisfactory performance in terms of accurate emotion detection. We have also shown how techniques such as task-specific pre-training, dimensionality reduction and quantization can be employed to boost performance or trade-off generalization capabilities and efficiency. In the future, we will investigate whether the use of different feature extraction techniques but also emotion categorization models, e.g., the Hourglass model, can further improve performance.

## Figures and Tables

**Table 1:** Emotion distribution in MELD. Data splits as reported in [58].

	Train	Validation	Test
Neutral	4710	470	1256
Joy	1743	163	402
Surprise	1205	150	281
Anger	1109	153	345
Sadness	683	111	208
Disgust	271	22	68
Fear	268	40	50

**Table 2:** Sample utterances from the dataset with the real label in the second column, and the predicted label in the third column.

Utterance	Real Label	Prediction
Ohh, that’s a good one.	Joy	Joy
Someone on the subway licked my neck! Licked my neck!!	Disgust	Anger
Bob. Bob! Bob!!! What the hell are you doing?!	Surprise	Anger
Oh my good God.	Disgust	Joy

**Table 3:** Emotion distribution in IEMOCAP.

	Train	Validation	Test
Frustrated	1210	258	381
Neutral	1080	244	384
Angry	749	184	170
Sad	764	75	245
Excited	520	222	299
Happy	376	128	144

**Table 4:** Weighted F1 Score on test set. The results are averaged over three runs.

RoBERTa		MobileBERT		BERT-Medium		BERT-Tiny	
BP	0.615	BP	0.609	BP	0.591	BP	0.574
Hidden	0.587	Hidden	0.568	Hidden	0.578	Hidden	0.519
Linear	0.586	Linear	0.531	Linear	0.566	Linear	0.507
ELM	0.565	ELM	0.537	ELM	0.532	ELM	0.508
OSELM	0.569	OSELM	0.54	OSELM	0.524	OSELM	0.486

**Table 5:** Comparison, in terms of weighted F1 Score, with the the variants that are pre-trained on IEMOCAP.

	RoBERTa		MobileBERT		BERT-Medium		BERT-Tiny	
	Regular	Pretrained	Regular	Pretrained	Regular	Pretrained	Regular	Pretrained
Hidden	0.587	0.598	0.568	0.589	0.578	0.54	0.519	0.544
Linear	0.586	0.598	0.531	0.584	0.566	0.524	0.507	0.523
ELM	0.565	0.586	0.537	0.56	0.532	0.51	0.508	0.531
OSELM	0.569	0.591	0.54	0.561	0.524	0.505	0.486	0.505

**Table 6:** Comparison, in terms of weighted F1 Score, with the variants to which PCA reduction is applied.

	Linear	Hidden	ELM	OSELM
BERT-Tiny 128	0.507	0.519	0.508	0.486
BERT-Tiny PCA 64	0.496	0.514	0.509	0.472
BERT-Tiny PCA 32	0.482	0.511	0.499	0.453
BERT-Medium 512	0.566	0.578	0.532	0.524
BERT-Medium PCA 128	0.538	0.567	0.53	0.506
BERT-Medium PCA 64	0.534	0.553	0.526	0.495

**Table 7:** Performance when a single user data is used. Results in terms of weighted F1 Score for all the users, varying the amount of data used, are reported.

	All	500	200
Phoebe	0.475	0.479	0.431
Joey	0.522	0.525	0.498
Ross	0.497	0.477	0.447
Rachel	0.495	0.472	0.418
Monica	0.474	0.46	0.437
Chandler	0.474	0.458	0.477

**Table 8:** Feature extraction on Jetson Nano.

	TINY		MED	
	FP16	FP32	FP16	FP32
Max-N	17.2 (1.1)	17.1 (0.7)	24.28 (0.7)	24.02 (0.7)
5W	33.96 (1.5)	33.59 (1.5)	41.62 (1.8)	42.13 (0.3)

**Table 9:** Feature extraction on smartphone.

	TINY			MED		
CHIPSET	FP32	FP16	INT8	FP32	FP16	INT8
Snapdragon 765G	2.6 (0.5)	2.6 (0.5)	1.9 (0.3)	88.0 (4.5)	85.0 (2.8)	32.8 (0.5)
HiSilicon Kirin 655	12.3 (0.7)	12.1 (0.5)	9.6 (0.8)	460.6 (0.9)	456.8 (1.7)	242.5 (0.7)

**Table 10:** Training of the linear classifier on Jetson Nano.

Working Mode	# Features	Z=200	Z=500	Z=1300
5W	32	44.4 (2.8)	103.8 (6.7)	328.2 (12.7)
	64	53.4 (3.1)	149.9 (10.2)	475.5 (21.1)
	128	47.2 (2.3)	227.8 (12.2)	799.3 (43.9)
	512	78.4 (3.9)	264.5 (3.0)	1909.6 (165.4)
Max-N	32	28.3 (2.1)	69.8 (6.2)	199.8 (12.5)
	64	33.3 (2.9)	96.7 (9.9)	304.4 (26.5)
	128	31.5 (0.9)	145.7 (14.9)	525.4 (40.4)
	512	52.5 (2.2)	179.6 (14.9)	1284.3 (117.4)

**Table 11:** Training of the linear classifier on smartphones.

CHIPSET	# Features	Z=200	Z=500	Z=1300
Snapdragon 765G	32	291.4 (6.12)	749.5 (5.85)	1847.4 (20.16)
	64	255.1 (8.51)	760.9 (33.46)	1964.8 (163.69)
	128	331.5 (7.96)	766.5 (73.2)	2119.8 (90.45)
	512	447.2 (8.56)	1376.2 (66.27)	2596.8 (138.59)
HiSilicon Kirin 655	32	378.27 (27.40)	943.8 (28.82)	2461.4 (40.94)
	64	413.8 (28.98)	1033.8 (28.07)	2723.3 (34.65)
	128	488.1 (31.38)	1224.9 (31.12)	3230.9 (28.82)
	512	1103.1 (28.90)	2778.9 (31.16)	7250.3 (36.28)

## Data Availability

Publicly available datasets were used. The original papers introducing the datasets were cited.
